# “There is always a waitlist”: The experiences of perinatal women randomized to a waitlist condition in a trial evaluating a novel online self-directed intervention for perinatal anxiety

**DOI:** 10.3389/frhs.2022.957368

**Published:** 2022-08-19

**Authors:** Amanda J. Kristjanson, Madison P. Hardman, Kailey E. Penner, Megan E. Gornik, Teaghan A. M. Pryor, Sarah K. Petty, Gillian M. Alcolado, Patricia Furer, Kristin A. Reynolds

**Affiliations:** ^1^Department of Psychology, University of Manitoba, Winnipeg, MB, Canada; ^2^Department of Clinical Health Psychology, University of Manitoba, Winnipeg, MB, Canada; ^3^Department of Psychiatry, University of Manitoba, Winnipeg, MB, Canada

**Keywords:** mental health, mental health services, waitlist experiences, perinatal, anxiety, qualitative, thematic analysis

## Abstract

**Background:**

Pregnant and postpartum women are at a heightened risk for the development or worsening of mental health problems, with elevated rates of mood and anxiety disorders noted across studies. Timely access to mental health supports is critical during the perinatal period (spanning pregnancy to 1 year postpartum), to mitigate potential negative impacts on mother and child. In general adult populations, a small body of research has highlighted the association between being waitlisted for mental health services with a deterioration in mental health. Given the influx of changes experienced in the perinatal period, this population may face unique challenges around being waitlisted. There is a lack of research exploring the experiences of perinatal women waitlisted for psychological services. The current study seeks to understand the experiences of perinatal women randomized to the waitlist condition of a randomized controlled trial.

**Methods:**

*N* = 20 participants (4 pregnant, 16 postpartum) from Central Canada who were enrolled in a novel online self-directed intervention for perinatal anxiety completed a virtual qualitative interview concerning their experience during the 6-week waitlist period for this randomized controlled trial. Interviews were audio-recorded, transcribed, and analyzed according to reflexive thematic analysis.

**Results:**

Seven main themes were identified, depicting the waitlist experiences of perinatal participants: (a) “There is always a waitlist” (sub-themes: service availability, need to seek out services pre-emptively); (b) Timing of support access is vital during the perinatal period (sub-themes: prenatal, postpartum); (c) Responses to being waitlisted (sub-themes: disappointment, neutral, relief, “there's probably somebody that needs it more than I do”); (d) Identification of helpful supports during the waitlist period (sub-themes: formal supports, informal supports); (e) Connections with research team (sub-themes: communication, resource provision); (f) Impact of waitlist experience on desire to start program (sub-themes: excitement, “out of sight, out of mind,” nervousness); and (g) Improving the waitlist experience (sub-themes: communication, resource provision, triaging).

**Discussion:**

Findings highlight the need for timely access to mental health supports during the perinatal period and offer several recommendations for improving the waitlist experience, including providing more frequent waitlist status updates, providing more direct access to intermediate interventions, and triaging patients based on clinical need.

## Introduction

It is well established that internationally, waitlists are considered a normal part of healthcare culture (1, 2). Within the context of the COVID-19 pandemic, there have been global reports of delayed procedures and elongated wait-times for medical treatments across populations (3). Although some information is known about the effects of waitlists for patients awaiting certain healthcare services (1, 4–6), there is a lack of empirical knowledge regarding the waitlist experience from the perspective of patients awaiting mental health services (7–10). Even less is known about what the waitlist experience is like for populations particularly at risk for the development or worsening of mental health problems, including pregnant and postpartum individuals (11–19).

During the perinatal period (spanning pregnancy up to 12 months following childbirth), there are numerous changes occurring within physical, emotional, hormonal, relational, and lifestyle domains that can be associated with elevated risk for mental health problems (11, 13, 17–19). Amongst these, perinatal anxiety is particularly common (20). Prior to the COVID-19 pandemic, ~10–20% of perinatal people experienced anxiety and related mental health problems (20–22); however, these statistics likely under-represent the prevalence of mental health problems during the perinatal period. One factor that can contribute to the under-reporting of perinatal anxiety is low help-seeking rates amongst perinatal individuals experiencing mental health problems (23–25). Prior qualitative research has suggested several reasons why perinatal people experiencing anxiety may not seek help for their mental health concerns, including uncertainty about their new roles as mothers, unrealistic expectations and feelings of inadequacy, and fear of looking like a bad mother (23). Further to this, studies have found that pregnant and postpartum individuals may have limited knowledge about their anxiety and/or depression symptoms and treatment options, which can further impede upon help-seeking (23, 24).

With the arrival of the global COVID-19 pandemic, mental health problems within perinatal populations have worsened (14, 26–28), with prevalence rates of prenatal and postpartum anxiety rising to 42% (29) and 61% (30), respectively. Public health restrictions have significantly altered or disrupted routine perinatal care, often forcing perinatal individuals to attend appointments on their own, which has contributed to additional feelings of stress in this population (14, 31). Further to this, pandemic related restrictions have reduced opportunities for family or peer support, thereby exacerbating feelings of isolation commonly experienced by this population (23, 26, 32–34). Findings by Vigod et al. (35) illustrate an increase in postpartum mental health service use during the first 9 months of the pandemic in Ontario, Canada, in particular for anxiety, depression, and substance use disorders. Perinatal mental health services frequently have additional limitations around availability and accessibility, such as stigma around accessing mental health services as well as a limited number of specialized service providers (22, 24, 36); given this, an increased demand for services coupled with a decrease in service availability may translate into elongated wait times. The long-term effects of this may have profound consequences, as it is well documented that without effective and timely access to treatment, perinatal anxiety can persist into early parenting years and beyond (23, 24, 37–39). Further to this, when present during pregnancy, increased maternal stress response may disrupt fetal development and increase the risk of preterm labor and spontaneous abortion (40, 41). Untreated perinatal anxiety may also lead to cognitive impairments in baby and long-term behavioral and emotional problems in the child (42–44).

At present, there are no research studies that have explored the experiences of perinatal people waitlisted for mental or physical health services or research trials. Few studies have explored the experiences and impacts of being waitlisted for mental health services or research trials within non-perinatal adult populations. Among non-perinatal populations awaiting medical or mental health procedures or services, qualitative and quantitative research has found that waitlisted individuals often reported a deterioration in their mental health, including increased anxiety, during the waitlist period (4–7). Further to this, adult waitlist populations expressed a need to receive treatment (medical or mental health) sooner. Previous studies have additionally found that individuals benefit more from medical or mental health treatments if they are received in a timely manner (5, 7, 10). Previous qualitative studies indicated that many individuals feel disappointment about being waitlisted for medical procedures and mental health services (8–10), while others reported feelings of abandonment and decreased self-esteem in response to being waitlisted (9). However, qualitative studies have also found that participants may express gratitude for being placed on a waitlist, particularly if they feel anxious or require time to prepare themselves for an upcoming treatment or procedure (7, 10). In prior qualitative studies, adult participants awaiting medical and mental health procedures and treatments have reported using a number of strategies to help them to cope with the waitlist experience, including keeping busy with work; cleaning; spending time in nature; exercising regularly; maintaining regular contact with family, friends and pets; utilizing self-help materials; obtaining social support from a general practitioner; and watching television or listening to music (7, 10). Previous qualitative studies that have sought to identify how the waitlist experience might be improved identified four recommendations: (a) periodic updates from healthcare providers, (b) transparency regarding the prioritization criteria, (c) estimates for the length of wait, and (d) intermediate interventions to help participants through the wait (5, 6).

Given the numerous and rapid changes occurring throughout the perinatal period, and the potential influx in mental health problems that can occur during this time, the effects of being waitlisted for mental health services may be profound and warrants examination. The purpose of this study was to investigate the experiences of pregnant and postpartum women struggling with anxiety who have been waitlisted for an online self-directed mental health intervention in a randomized controlled trial. Our research adds to the limited body of research that currently exists around mental health waitlist experiences, while considering the unique implications for perinatal populations.

## Materials and methods

This study was part of a larger randomized control trial titled *Evaluating a Self-Directed Cognitive Behavioral Therapy E-Health and M-Health Program for Anxiety in Pregnancy and Postpartum During COVID-19* (ClinicalTrials.gov, identifier: NCT04844138). Institutional and hospital review ethics board approvals were received prior to the commencement of this work. The aims of the larger study were to examine the impact of an online self-directed Cognitive Behavioral Therapy (CBT) program for pregnant and postpartum individuals residing in Central Canada experiencing anxiety.

### Population and sample

#### Overcoming perinatal anxiety online self-directed CBT program

Participants were recruited for the Overcoming Perinatal Anxiety (OPA) online self-directed CBT program via advertising through community organizations, hospitals, clinics, and university settings, in addition to social and mainstream media. Eligibility criteria included age (18 years of age or older), being pregnant or within 12 months postpartum, experiencing self-reported symptoms of anxiety, living in Manitoba, Canada, English language fluency, and Internet access. Individuals who were experiencing current substance use disorders, diagnoses of schizophrenia or bipolar disorder, and current suicidality were excluded from the study. Interested participants who were engaged with a counselor or psychologist and those who were on stable medication regimes at the time of the study were included, however, these variables were considered in quantitative data analysis within outcome research in the overarching study.

Interested participants were first asked to complete a screening interview with the research coordinator or a designated research assistant to determine initial eligibility. Individuals who did not meet the inclusion criteria were provided with a list of alternative resources. For participants who were deemed eligible upon screening and completed written consent, a clinical symptom interview [Mini International Neuropsychiatric Interview (MINI); Adult Standard MINI 7.0.2; (45)] was scheduled with a trained member of the research team. If participants endorsed symptoms within the DSM-5 (46) as assessed on the MINI 7.0.2 (with a primary focus on anxious and depressive symptoms) they were considered eligible. Individuals who met criteria for current substance use disorder, bipolar disorder, or schizophrenia during the clinical symptom interview were excluded and provided with a list of alternative resources. If participants exhibited current suicidality during their screening or clinical symptom interview, the team member conducting the interview immediately consulted with a clinical psychologist on our team to ensure participant safety and to determine participant eligibility.

If participants were deemed eligible following the clinical symptoms interview, they were then randomized into the CBT online self-directed program or waitlist control condition. Participants randomized to the CBT program condition began treatment immediately, while those in the waitlist condition waited 6 weeks before receiving access to the online program. Following communication of waitlist assignment and completion of baseline quantitative measures, waitlisted participants received a check-in email from the research coordinator at week three. The purpose of this check-in was to determine whether waitlisted participants were experiencing distress and to remind them that they could seek additional services during the waitlist period. A list of alternative resources was also sent to them at this time. Participants in both conditions had access to their usual care throughout the study.

#### Waitlist condition

Participants for the present study were recruited from the OPA waitlist control condition. Participants in the waitlist condition completed a questionnaire battery at Week 1 (to collect baseline data at the start of the waitlist period) and Week 6 (to be compared to the post-program assessment for those in the CBT program condition, and to capture any changes in their symptoms since the start of the waitlist period). Waitlisted participants were invited on a rolling basis to participate in a semi-structured qualitative interview at the end of the waitlist period. 20 participants completed interviews (n = 20), with data collection ceasing upon the achievement of theoretical saturation, the point at which no new thematic information is generated upon analysis (47).

### Study design

In keeping with previous research that sought to explore the waitlist experiences of patient populations awaiting medical procedures or mental health services (e.g., surgery, mental health support) (1, 4–10, 48), a qualitative design was used to describe the waitlist experiences of expectant and new mothers experiencing anxiety during the perinatal period. To understand the nuance and complexities of participants' experiences, we followed Braun and Clarke's (49, 50) reflexive thematic analysis approach along with Tracy's (51) criteria for qualitative rigor. Given the novelty of this research, it was important for us to remain grounded in participant data in an inductive manner, to understand the unique experiences and insights participants had about their time spent on the waitlist and to explore common themes across multiple participants' experiences. Quantitative data obtained from participants was used to contextualize qualitative findings.

### Procedure

Virtual semi-structured qualitative individual interviews were completed by trained research team members. These team members were part of the overarching OPA study; select interviewers held additional roles within the study and therefore had ongoing communication with participants beyond the interview. Interviews lasted ~30 mins and were audio-recorded, transcribed, de-identified, reviewed for accuracy, and uploaded into *NVivo* for thematic analysis.

To facilitate a deep exploration of the topic and to receive a rich description of participants' experiences, interview questions were open-ended in nature. Examples of interview questions included the following: “*What has your experience been like as a waitlist participant in this study?,” “How have you been feeling during this time?,” “What resources, supports, or services have you accessed (if any) while you have been on the waitlist?,” “How have you coped with challenges that may have come up while you have been on the waitlist?,” “How are you feeling about starting the OPA program?,” “How could your waitlist experience have been improved?*.” Probes were included depending on participant responses to increase depth.

### Questionnaires

####  Demographic questionnaire

Prior to beginning the CBT online self-directed program, both program and waitlist condition participants completed a demographic questionnaire. This included information on age, level of education, occupational status, marital status, ethnicity, perinatal status (i.e., pregnant or postpartum), weeks gestation if pregnant, age of infant if postpartum, physical health in pregnancy, number of pregnancies, number of children, neonatal loss, termination, mother-baby bond, physical and mental health throughout the lifespan, past experiences with depression and anxiety, and past and present mental health help-seeking experiences, with a specific focus on medication and psychological treatment. Additional information regarding challenges with pregnancy, miscarriages, labor and delivery, and postpartum mother and infant health was collected to characterize our sample. A subset of our demographic data was included in the present study to contextualize our sample.

#### Edinburgh Postnatal Depression Scale

The Edinburgh Postnatal Depression Scale (EPDS) is a 10-item self-report measure assessing symptoms of postnatal depression on a 0 to 3 scale with a range of verbal response options for each question (52). Contrary to its name, it is considered acceptable for use in both pregnant and postpartum populations (53). The EPDS is scored by summing participant responses using Likert-scale anchors. It exhibits satisfactory psychometric properties, is sensitive to change (52), and has excellent test-retest reliability (54). Item 10 (suicidality measure) was removed as individuals with current suicidality were screened out of our study during the screening and clinical symptom interviews. The EPDS was administered to waitlisted participants as part of the larger OPA study at the start of the waitlist period, end of the waitlist period, at the end of each OPA module, after completion of the CBT program, and 1-month after program completion to assess changes in depression symptoms across time.

####  Perinatal Anxiety Screening Scale

The Perinatal Anxiety Screening Scale (PASS) is a 31-item self-report measure assessing symptoms of anxiety during the perinatal period on a 0 to 3 scale, with verbal response options ranging from *not at all* (0) to *almost always* (3) (55). The PASS is scored by summing participant responses using Likert-scale anchors. The scale has excellent reliability, adequate test-retest reliability, and strong evidence of convergent validity (55). The PASS was administered to waitlisted participants as part of the larger OPA study at the start of the waitlist period, end of the waitlist period, after completion of the CBT program, and 1-month after program completion to assess changes in anxiety symptoms across time. A shortened version of the PASS was also administered at the end of each OPA module.

### Data analysis

#### Qualitative data

Three members of our research team engaged in the six-phase approach of reflexive Thematic Analysis to ensure trustworthiness of qualitative data (49, 50). *NVivo* qualitative research software was used to assist with data organization. Interviews were analyzed as follows. First, researchers familiarized themselves with the collected data through repeated readings and engaged in initial note taking. Initial codes were then produced from the data. Researchers coded for as many potential themes as possible and endeavored to code extracts of data inclusively, as is recommended by Braun and Clarke (50). Following, researchers considered how codes might combine to create overarching themes. During the next phase, researchers refined themes to create a “thematic map” (50). Some themes were discarded while others collapsed into each other, creating one broader theme. Others still required breaking down into smaller, separate themes. Once a satisfactory thematic map was created, researchers further defined and refined themes to be presented in the final analysis, whilst continuously considering how each theme fit into the broader ‘story' being told (50).

### Reflexivity and rigor

To ensure richness in rigor and trustworthiness of research, several procedures were followed in keeping with those outlined by Braun et al. (47), Braun and Clarke (49), and Tracy (51). The research team brought with it a broad range of personal and professional experiences in perinatal mental health. Undergraduate and Graduate Psychology students conducted interviews under the guidance of a clinical psychologist with specialized expertise in the field. Additionally, the psychologist assisted in the development of the qualitative thematic framework and remained available for consultation throughout analysis. These differing lenses therefore impacted the resulting analysis, requiring researchers to engage in a reflexive collaboration and exchanging of ideas (47). All members of our research team endeavored to engage reflexively in their analyses, recognizing how their own experiences and potential existing relationships with participants within the context of the overarching study might shape their interpretation of the data [(51); p. 841]. In keeping with Braun and Clarke's (49) reflexive thematic analysis procedures, the research team endeavored to center “researcher subjectivity, organic and recursive coding processes, and the importance of deep reflection on, and engagement with, data” (p. 593). Researchers therefore coded independently and then came together to discuss their findings and revise the thematic framework accordingly. The collaborative nature of the research methods applied throughout analysis also served to increase the credibility of this study. As new themes emerged, and as codes expanded and collapsed, researchers continued to revisit the coding framework. Further, in keeping with Tracy's (51) recommendations for ensuring richness in rigor, a coding journal was kept as an audit trail for all coding decisions.

## Results

### Quantitative data

Descriptive statistics were calculated and included to contextualize the current study sample. Table 1 outlines the sociodemographic characteristics of our waitlist sample at baseline. Among our participants, the mean age was 33.75 years. With regards to perinatal status, 20% of respondents were pregnant and 80% were postpartum. In this sample, all respondents identified as female (100%), the majority identified as European (60%), nearly all participants had completed a university or college program (90%), and all were married or in a common law relationship at the start of the waitlist period (100%). The majority of the sample (80%) reported experiencing anxiety or depression in their lifetime. Some respondents (30%) disclosed that they were taking medication for treatment of a mental health problem, and 25% of respondents disclosed that they were receiving psychological support from a counselor or psychologist. Table 2 outlines waitlisted participants' EPDS and PASS scores at baseline and end of the waitlist period.

**Table 1:** Sociodemographic characteristics of waitlist participants at baseline (*N* = 20).

**Characteristics**	***M* (SD) or *n* (%)**
**Perinatal status**	
Pregnant	4 (20%)
Postpartum	16 (80%)
**Age (years)**	33.75 (4.12)
**Gender identification**	
Female	20 (100%)
**Education**	
Some college/trade school	1 (5%)
Some university	1 (5%)
Graduated college/trade school	3 (15%)
University undergraduate degree	8 (40%)
University graduate degree	7 (35%)
**Ethnicity**	
Asian	2 (10%)
Armenian	1 (5%)
European	12 (60%)
European and French Canadian	1 (5%)
European and Indigenous	2 (10%)
Indigenous	1 (5%)
Latin, Central, South American	1 (5%)
**Marital status**	
Common law	2 (10%)
Married	18 (90%)
**Previous anxiety or depression in lifetime**	
Yes	16 (80%)
No	4 (20%)
**Mental health medication use**	
Yes	6 (30%)
No	14 (70%)
**Undergoing psychological treatment**	
Yes	5 (25%)
No	15 (75%)

**Table 2:** EPDS and PASS scores at baseline and end of waitlist period (*N* = 20).

	**EPDS scores**		**PASS scores**	
	**Baseline**	**End of** **Waitlist**	**Baseline**	**End of** **Waitlist**
Mean	10.85	10.2	39.85	34.7
Standard deviation	4.78	5.60	19.64	18.95
Range	15.0	19.0	77.0	65.0
Minimum	4.0	1.0	10.0	11.0
Maximum	19.0	20.0	87.0	76.0

### Qualitative data

Seven main themes and related sub-themes regarding perinatal women's experiences on a waitlist for a randomized controlled trial emerged from the analysis of qualitative interview transcripts: (a) “*There is always a waitlist”* (sub-themes: service availability, need to seek out services pre-emptively); (b) *Timing of support access is vital during the perinatal period* (sub-themes: prenatal, postpartum); (c) *Responses to being waitlisted* (sub-themes: disappointment, neutral, relief, “there's probably somebody that needs it more than I do”); (d) *Identification of helpful supports during the waitlist period* (sub-themes: formal supports, informal supports); (e) *Connections with research team* (sub-themes: communication [facilitators; barriers], resource provision); (f) *Impact of waitlist experience on desire to start program* (sub-themes: excitement, “out of sight, out of mind,” nervousness); and (g) *Improving the waitlist experience* (sub-themes: communication, resource provision, triaging). Findings are presented below with additional participant quotations found in Table 3.

**Table 3:** Additional qualitative responses for each main theme and sub-theme.

	**Main theme: There is always a waitlist**	
**Sub-theme**	**Direct quote (Participant ID)**	**In-text identifier**
Service availability	“I had called other psychologists, and it was just such a long, long wait. So this is the first one that I could access almost right away, even though I ended up being on the waitlist, it was still something that I could do in terms of dealing with the anxiety” (Participant 4).	1
Need to seek out services pre-emptively	“I just, I wish that there were more programs. I wish it was something that you could have acknowledged, especially because this is my second child, especially knowing what I dealt with the first time. I wish that we could almost be placed on waitlists in pregnancy. Be offered those resources from our doctors like, hey, I know you dealt with this last time, let's put you on a waitlist now so that you're fast tracked by the time that you do give birth and you're not sitting at home waiting” (Participant 16).	2
**Main theme: Timing of support access vital during the perinatal period**		
Prenatal	“I found that…as I got closer to giving birth, the anxiety, you know, sort of amped up with—we had, you know, I had a fetal assessment at thirty five weeks and he was small. And what did that mean? And then my blood pressure started going up and then that was scary as well… It wasn't that I wasn't able to function, but it preoccupied my mind quite a bit. I started being really nervous about giving birth and what that would look like, particularly because I felt like we found out he was small and that then sort of threw me for a loop because prior to that, you know, everything was normal” (Participant 8).	3
Postpartum	**“**So the earlier you are, it's like that's the hardest place. It's like that's where it would be the most value. So it's not to say that once I do have access to the program, it won't give me value. I just feel like the sooner the better…in the last month, I find [that] yes, I'm still struggling, but I do find that I have a lot more moments of clarity these days and so my highs and lows are getting a little bit more level, if that makes sense…I'm just getting that better sense of freedom. I have more time to kind of connect with myself and be my own person. And that definitely helps…I know that I will find a lot of value in the program once I have access to it. But I do wish I had it sooner because this is the hardest part.” (Participant 1)	4
**Main theme: Responses to being waitlisted**		
Disappointment	“Yeah, I mean, there's always disappointment when you can't start something right away, but at the same time I kind of checked in and was like, you know what? My anxiety is not to a point where I'm so concerned that I feel like I need to [start the program] right away. So I felt comfortable with being on the waitlist, but also just grateful that I'll be able to take part in the study. So. [pauses]. Yeah, initial like…disappointment, but then also just grateful that'll be able to take part eventually…” (Participant 12).	5
Neutral/Relief	“…I was just excited to be able to participate, so knowing that I had to wait was totally fine” (Participant 20).	6
“There's probably somebody that needs it more than I do”	“At first I just thought it was like, oh, I'm not like on the severe—like, I kind of thought maybe it wasn't random and I wasn't like [in] as dire need [of] the support and that I think I had vocalized that like I did already have my individual supports and like it wasn't going to make or break it, but like I would really like to go through this experience” (Participant 10).	7
**Main theme: What was helpful during the waitlist period**		
Formal supports	“A lot of it is also, I think, reassurance. I think there's a lot of mom guilt that I go through. Sometimes I question whether I'm good enough as a mom, especially in the day when I'm just having, you know, really challenging moments with the baby. And I just, yeah—and I think [the nurse practitioner] plays this big role of just reassuring me like, I'm doing my best. I am a good mom—and it—it helps to hear that—and also just having another human interaction because sometimes it is very isolating” (Participant 19).	8
Informal supports	“I'm just doing my best to also talk to friends and family. Last time I was very, I don't want to say embarrassed, but there was that whole stigma around it [which] was a lot harder to deal with. Whereas now because I've already talked to people about it in my life, I don't feel that same kind of hesitation to discuss it with them. So that's also been a thing that helps” (Participant 1).	9
**Main Theme: Connections with Research Team**		
Communication		
Facilitators	“I think one of the biggest benefits was that I just didn't have to talk to anyone in-person. When I initially reached out, [I just sent] an email and [then never had to] look back until the next step started. So, even though I feel a bit of anxiety before coming on a Zoom meeting, I think just knowing that it's booked and then we just do it [is helpful to] keep carrying forward. But I do think that having a video call for me is a bit easier than just talking on the phone. So, I think the accessibility has been helpful for me” (Participant 20). “I think just allowing myself more time to think about my response and craft it a little bit rather than thinking on the spot is just easier for me. So [email communication] was good” (Participant 5).	10 11
Barriers	“…Like through the camera, there's so much technical difficulties and things that happen that I just feel like more anxiety with that, you know, on top of…on top of my already anxiety issues. So like just getting this meeting going today, I was like, oh my gosh, my computer, it's like doing an update, and I can't get my phone to go on. And I was just like, I don't even know if I'm going to make it [laughing, but stressed tone]. So just face to face is just so much easier [laughing]” (Participant 11).	12
Resource Provisions	“I did look at the list and I felt like a lot of it was not geared to what I was experiencing, especially the crisis [lines]—Like, I haven't felt like in the last year that I've ever had a moment of crisis where I felt like I needed help in that exact moment, or that something would have been so severe that I needed help in that exact moment. So I felt like most of [the resource list] was not applicable…it's [for] someone that's in distress or crisis—in crisis or in psychosis or whatever you want to call it. But it's a major event, in my opinion. And so they're talking the person down or directing them for whatever their needs are” (Participant 9)	13
**Main Theme: Impact of Waitlist on Desire to Start Program**		
Excitement	“It's just I—like I want to feel like myself again. So I'm, like, eager to get started. Because even just like, because I do find like depression and anxiety are different, and so it feels familiar, but also unfamiliar and yeah, I'm just like excited to not feel this way” (Participant 1).	14
“Out of sight, out of mind”	“If you forget about it a little bit, [the check-in email] kind of gives you like that, Oh, yeah, I'm on the wait list and it's still happening… [So] I did kind of get lost in time there. But once I got the email, I was like, Oh right, it is happening.” (Participant 17)	15
Nervousness	“I think logically, my brain was really looking forward to it and having strategies, but my heart who doesn't like to deal with the feelings was feeling nervous about it. Like, what will this look like? What will this bring up? …So that was how I felt. I was like, Oh yay, it'll be great to get some strategies on this, especially because there's so many different things to be anxious about. Yeah, but the other part was like, Uh-Oh, what happens when I can't get out?” (Participant 8).	16
Communication	“So, I mean, the—the mind is susceptible to what's in front of you, right? So you could…[deep breath] for people that are on the waitlist, you could do a, not necessarily a reminder, but a sneak peek to keep people's—their appetite wetted, right? Here's some of the things you're going to learn. Here's, you know, some of the, you know, and you're not going into detail about the modules, but it's like, you know, this is—this is what you have to look forward to. This is what, you know, again, not about this is how it's going to happen and all the rest of it—but it's fluffy, right? Almost like an advertisement saying, hey this is, you know, we're, you know, hey, only 4 weeks to go or only or, you know, here's the things you have to look forward to” (Participant 3).	17
Resource Provision	“I think that the good thing about like something that's like self-directed like this is it's something that you could do during when you're on a waitlist right? Because really in the real world kind of thing…there's not usually like a cap on how many people that can read something at one time right? So if somebody is placed on a waitlist, then this is something that maybe that can like um help them in the meantime kind of thing. That's where I can see this you know working in the grand scheme of things.” (Participant 2)	18
Triaging	“I think if I was [in an acute phase of panic or anxiety], offering additional supports in that wait period [would be helpful]. And I know we got the resource list, but—I found it helpful to talk to a psychologist when I was having lots of symptoms of panic and anxiety last time, and so even just setting someone up with an appointment right away so that you can also gauge their—I don't know- how bad it is, because I would say mine was like a ten on a scale of ten being the worst. So. Yeah, this time it's like a two, if that, so I'm like, I'm just chillin waiting for the thing to start, whereas yeah, if I were to speak to the other end of that experience, it would be different” (Participant 18). “…I'm wondering if there was another mom that just felt like she needed to access [the program] right away if there's an option to follow-up, to say, if there was a cancelation or if someone drops, can I take their spot? Like if [someone's] anxiety gets worse because they have to wait to access the modules, if there's a way that they can contact you guys just to say, “is there any way that I can get in early” or something like that?” (Participant 12)	19 20

#### There is always a waitlist

Participants spoke of not being surprised to find themselves on the waitlist, indicating that this has been a frequent experience for them when seeking both perinatal and non-perinatal medical and mental health services in the past. Participants spoke of various factors affecting their ability to find and access services. Accordingly, two main sub-themes emerged: service availability and a need to seek out services pre-emptively.

##### Service availability

During the unique time of living through a global pandemic, some participants spoke of how COVID-19 had impacted their waitlist experience, both positively and negatively. In some instances, the presence of waitlists increased because of the reduced availability for in-person services. In these cases, women often felt the onus was on them to self-identify symptoms or manage physiological and mental health problems they might be experiencing, as they were having reduced contact with their regular healthcare providers. In other cases, participants expressed being able to access services more easily given the increase in remote services being offered and, consequently, a program's ability to increase its capacity limits. One participant discussed being able to gain access to a remote prenatal course that, had it been offered in-person, she would have otherwise been waitlisted for. Some participants also spoke of finding remote services easier to access because it meant not needing to find transportation or childcare in order to attend in-person appointments.

Participants who were particularly impacted by the increased availability of remote services were those residing in rural or remote locations. While the increased offering of remote services during the COVID-19 pandemic often made it easier for participants to access perinatal services, in instances where face-to-face care was required (both before and during the COVID-19 pandemic), participants spoke of the demand for services frequently outweighing availability. Additionally, it was expressed that services were often canceled if providers were unable to gain access to the community, therefore further elongating wait times for services. Participants residing in rural areas also indicated that there was often a lack of specific perinatal or mental health providers; in such instances, services would have to be delivered by another provider who may not have specialized training with perinatal populations.

“*…I could go to the mental health place here, but it doesn't mean this person really has any training specific to mental health, like [pauses] people don't want to work up north. So they just kind of fill positions in with, like the best that there is and hope for the best”* (Participant 7).

The same participant indicated that in some instances, the hospital may be the only place rural and remote residents can go for mental health support and even then, a person might get turned away to make space for someone who is “*sicker”* (Participant 7). She, along with other participants residing in rural areas, also indicated that it can be especially difficult to access mental health services in rural locations because of reduced access to childcare supports (both family and professional services), which may hinder help-seeking efforts.

It was further expressed that even before being placed on a waitlist, the process of finding mental health services may not be straightforward; specifically, financial barriers were identified as impeding service accessibility. Many of the sliding scale mental health services offered target people below a certain income level, which can pose challenges for those earning just above this threshold who may still find it challenging to pay for services out of pocket. Some participants indicated that they had insurance coverage to see a counselor or psychologist; however, this coverage was often limited to a reduced number of sessions, making participants feel as though it would be impossible to tackle their mental health issues in the limited amount of time available to them.

“*…you get eight sessions and then you kind of move on, right?…You can unpack, but you can't get it all back in your bag. So it can be difficult”* (Participant 16).

Multiparous participants also experienced challenges with service availability while trying to access free or subsidized mental health services that had been helpful during a previous perinatal period. Oftentimes they were no longer eligible for the same service with subsequent pregnancies because they had already accessed the service once before and therefore, used up their allotted sessions. Whatever the cause of reduced service availability, participants mentioned sometimes selecting services not necessarily based on what would best align with their needs, but based on which service offered the shortest waitlist (Table 3, quote 1).

Additionally, one participant discussed a cyclical experience of being waitlisted for mental health services. Participant 2 expressed feeling in crisis and the “*pivotal moment*” of mustering up the courage to seek help, only to be waitlisted for services. She then would find other ways of coping so that by the time it was her turn to receive the service, she was no longer in crisis and might not be as receptive to receiving help. “*…life goes on, right? So you have to deal with it until kind of something else really bad happens.”* (Participant 2). Having not necessarily dealt with the root cause of the issue, the participant indicated the cycle would then repeat itself.

##### Need to seek out services pre-emptively

In order to avoid long waitlists, participants spoke about needing to seek out mental health services preventatively; this knowledge was usually based on previous experiences of being waitlisted for similar services. For example, multiparous participants acknowledged that they were more likely to develop perinatal anxiety or depression in the current perinatal period if they had experienced mental health problems in earlier pregnancies or postpartum periods. Expecting to be waitlisted, participants therefore sought services in anticipation of need.

“*…because we know that through other things that, like it's always a waitlist, right? [Laughs]. So you kind of want to make sure you're in with somebody, or start doing something before things get bad, because once things get bad and then you try to get help, then you're waitlisted [laughs] usually”* (Participant 2).

One participant expressed a desire for care-providers, knowing the client's history and in anticipation of long waitlists, to be the ones to proactively make arrangements for treatment early on in the perinatal period, taking the onus off the individual themselves (Table 3, quote 2).

#### Timing of support access is vital during perinatal period

Part of participants' desire to proactively seek mental health services appertained to the importance of accessing timely support during the perinatal period, when many changes are happening at a rapid pace. The times participants identified as being most crucial for support were early in pregnancy and postpartum. As such, the two themes that were identified were prenatal and postpartum.

##### Prenatal

First time pregnancies, pregnancy health complications, and previous experience with miscarriages were identified as factors contributing to anxiety experienced during the prenatal period. Anxiety surrounding these concerns was heightened during the COVID-19 pandemic due to additional concerns of getting sick, changing public health restrictions, increased feelings of isolation, and reduced in-person access to regular perinatal care. Speaking to her pregnancy complications, one participant expressed feeling her anxiety decrease as gestational weeks increased. Another participant, however, indicated that after finding out that her baby was measuring small, felt her anxiety increased as her due date approached (Table 3, quote 3). Additionally, some participants identified a feeling of isolation during the perinatal period in which there was a felt expectation that, because pregnancy is considered a natural process, they should inherently know what to do. This further highlighted the need for timely availability of mental health services during the perinatal period to ensure that perinatal people feel supported during this period of uncertainty.

“*…through this whole process [pregnancy], it's kind of really opened my eyes as to how women in general are kind of left alone in pregnancy…It's actually very interesting to see the changes, I mean, with how women are treated in pregnancy, that's like, oh, you'll know what to do. You'll just push, we'll catch. It'll be fine. [laughing]”* (Participant 3).

##### Postpartum

Participants expressed feeling higher levels of anxiety early in the postpartum period. A range of factors were identified as contributing to their anxiety at this time. While some cited the COVID-19 pandemic along with ever-changing public health restrictions as the source of their anxiety, other participants identified shifting family dynamics (partner conflict, going from one to two children), lack of time for themselves (sleep, self-care), lack of support (childcare, household responsibilities), and hormonal changes as factors contributing to their mental health. Participants also spoke about feeling their anxiety decrease as the postpartum weeks increased and they were able to find more independent time for themselves; however, they expressed feeling like they would have benefitted more from timely access to support immediately following delivery (Table 3, quote 4). Several participants mentioned finding it difficult to identify what they were feeling. At times, this made it difficult for some participants to seek help.

“*...especially when you're in that space of anxiety, it's hard to reach out for help sometimes. It's hard to pull yourself out of that and even realize what's going on, especially when you're trying to figure out, like, is [this] the baby blues? Is this just normal?”* (Participant 1).

### Responses to being waitlisted

For those who did manage to seek help and found themselves waitlisted, the responses to being waitlisted varied, with four sub-themes emerging: disappointment, neutral, relief, and “*there's probably somebody that needs it more than I do.”* In most cases, participants did not express positive feelings about being waitlisted, but what distinguished responses appeared to be the participant's level of clinical need. Another way of responding was a more practical worry about how the timing of getting off the waitlist would align with the participant's delivery date. Amongst all sub-themes, there were participants who expressed feeling that knowing they were on the waitlist and would eventually receive treatment made the wait time feel more feasible.

#### Disappointment

A frequent response to being waitlisted was disappointment, which ranged from mild responses to stronger emotional reactions. Some participants expressed that while they were disappointed, knowing that they would eventually receive treatment made the 6-week wait time more manageable (Table 3, quote 5).

Participant 2 indicated that it came as no surprise to them to be waitlisted; **“***[laughs] I guess it was kind of expected.”* While expected, the same participant indicated that “*it sucks*” to be waitlisted, revealing a level of disappointment that aligned with other participant responses. Participants also spoke of the pivotal moment of being empowered to seek help, only to be waitlisted, and correspondingly deflated.

“*- like it's hard to reach out for help sometimes…then you're like, no, I do need help. You're kind of like, oh, great, I reached out, but now I don't get it”* (Participant 1).

#### Neutral

Neutral responses to being waitlisted were also common, particularly amongst participants who voiced less acute symptoms of distress. One participant indicated that she might have had a stronger response to being waitlisted had it been earlier in the perinatal period, but because she found herself later in the postpartum period, she did not feel as desperate to receive service.

“*…the fact that I had to wait 6 weeks didn't impact me a lot, because my anxiety was the most strong in the first, let's say, 3 weeks postpartum…and by [the time I was waitlisted] the worst of it had already kind of passed.”* (Participant 14).

Several participants expressed that feeling excited to simply be accepted into the program made it feel easy to wait the 6-weeks (Table 3, quote 6).

#### Relief

While some participants had neutral responses to being waitlisted, a few participants expressed more profound feelings of relief. For some, their relief was related to timing; these participants indicated that it was more convenient to delay their participation in the program given the circumstances of their personal lives, such as a career change. Similar to the participants whose responses were more neutral, several of these participants echoed that the feelings of relief of being accepted into the program outweighed any negative feelings about being waitlisted and made it feel more feasible to cope with having to delay their treatment. “*…at least I'm getting some sort of support and resource afterwards…”* Participant 6 said. One participant shared her perception that being waitlisted was a positive sign to her, that she was perhaps not as “*sick*” as she initially thought.

“*[Being waitlisted was] almost a relief—I felt like maybe I wasn't [pauses]…[sighs] it sounds weird, but as sick as I thought I was, maybe? So…[I wasn't] as anxious. I felt like, OK, I can manage this on my own, obviously I don't need [the] intervention* *right now* *kind of thing”* (Participant 4).

#### There's probably somebody that needs it more than I do

Despite knowing the selection process was random, in response to being waitlisted many participants spoke of a deeper impact of questioning their own self-worth. Several participants expressed thinking they weren't “*good enough for the program”* (Participant 1, 2), while others rationalized that their symptoms must have been considered not severe enough to warrant immediate intervention (Table 3, quote 7).

### Identification of helpful supports during the waitlist period

Participants had varying ways of coping with being waitlisted. Many identified their use of formal and informal supports while on the waitlist, with formal supports encompassing more organized, clinical resources and informal supports including self-guided or peer resources. Participants' past and present experiences with depression and anxiety often guided the types of resources they sought out. Participants who had previous experiences with depression or anxiety tended to favor strategies that worked well for them in the past and avoided those that had not.

#### Formal supports

Formal supports accessed during the waitlist period included speaking with a healthcare provider, receiving counseling or psychotherapy (both individual and family), educational classes, and medication. Many participants described that speaking with a healthcare provider, counselor, or psychotherapist was helpful in reducing their feelings of anxiety (Table 3, quote 8).

In addition to speaking with a professional, the other most commonly reported formal support was medication. While many participants who were on medication expressed finding it helpful, many also indicated that they would prefer not to be taking it and wanted greater access to other forms of treatment that would allow them to discontinue use.

“*…I wish there were doctors that didn't just push medication and tell you to be mindful, and then just be like, oh, 4 weeks from now, you don't feel better? Let's try something else. Like, I wish that that wasn't the way that it is, [but] it is. And that's unfortunate, but not a whole lot you can do”* (Participant 16).

#### Informal supports

Participants cited a number of informal supports that were helpful in managing or reducing feelings of anxiety during the waitlist period. These included self-guided resources, such as pregnancy applications (e.g., What to Expect app), self-help books and podcasts, and breathing/meditation techniques, in addition to social network support, which included relying on partners, family, and friends for support. When it came to peer supports, finding other mothers who had similar experiences seemed especially helpful.

“*…my best friend just had her second, so…we're both in the postpartum stage, and I made a couple of mom friends…So we've been keeping in touch and it's really nice—I feel like it's a safe group where we can be really vulnerable when our anxiety is spiking or [when] we're just having a tough day and it's not all rainbows and sunshine and we just need to talk to somebody about it. I [feel like] I'm really lucky that I have that support”* (Participant 12).

While many participants felt quite comfortable speaking with family and friends, in some cases, previous experiences with anxiety or depression seemed to mediate some participants' willingness to seek peer support (Table 3, quote 9).

### Connections with research team

Participants generally spoke positively of their interactions with the research team. Two main sub-themes emerged within this theme: communication (facilitators, barriers), and resource provision.

#### Communication

##### Facilitators

Participants expressed appreciation for the research team's “*empathetic, professional manner”* (Participant 1) and the ease in which they were able to communicate with the research team through virtual means (Table 3, quote 10). The majority of participants expressed that they felt they had received sufficient information ahead of time about the study. Many participants also expressed appreciation over the realistic timelines that were communicated with respect to the 6-week duration of the waitlist; this meant that participants knew when to expect to come off the waitlist, something that may have been different from previous waitlist experiences. The majority of participants also indicated they appreciated receiving communication via email (as opposed to telephone calls or other forms of communication), with some specifying they liked that it allowed them time to craft their responses (Table 3, quote 11). Participants were particularly appreciative of the check-in email that was sent at week three of the waitlist period, indicating that it was a nice “*reminder that you're not forgotten, that [the treatment program] is coming”* (Participant 6).

##### Barriers

While most participants felt at ease with the online format in which communication with the research team took place (e.g., email, videoconference), a minority of participants indicated they would have preferred more communication via telephone or face-to-face, with one participant identifying feelings of anxiety around connecting through a virtual format (Table 3, quote 12). Similarly, another participant indicated that virtual forms of communication were made more complicated by the presence of her older children at home, who were engaged in remote-learning during the COVID-19 pandemic and whom she also needed to attend to.

“*The only thing is if my kids are at home, it's impossible really. If they were here, they would just bomb the whole conversation—they would sit here and ask you questions and try to tell you what they did today. And really, it's going to be just difficult, 100 percent”* (Participant 15).

####  Resource provision

Additionally, to help facilitate access to formal supports during the waitlist period, the research team provided participants with a list of mental health and perinatal resources. While all participants expressed a general appreciation for this list, few people accessed the resources therein. Reasons for not utilizing the resource list included lack of time; feeling overwhelmed; not knowing which resources were most appropriate; and having other supports already in place.

While the thoroughness of the list was generally viewed as a positive thing by most participants, many identified that it could potentially be overwhelming, especially when they were already feeling mentally depleted.

“*I could have, should have sought out or used resources. I just didn't feel mentally capable. You know, when you're coming out of something, it's difficult to sort out… I think the bottom line for me [is that] when my anxiety is at a high, I get quite stuck in it and… you know, even if someone said to me, “Hey, you should go to this website,” I would give you five reasons why I would be unable to –”* (Participant 8).

Additionally, while both emergency and non-emergency resources were provided, some participants expressed that it was difficult to discern which resources were applicable to them and a few were under the impression that they could only access these resources if they were in crisis (Table 3, quote 13).

### Impact of waitlist experience on desire to start program

As their time on the waitlist came to an end, all participants expressed an eagerness to begin the program, with this level of excitement seeming to vary based on clinical need. A few participants also expressed mixed feelings about beginning the program. Three main sub-themes therefore emerged under this theme: excitement, “*out of sight, out of mind,”* and nervousness.

#### Excitement

All participants expressed feelings of excitement or eagerness to begin the treatment program (Table 3, quote 14). Many participants seemed hopeful about commencing the program and were looking forward to “*feeling like [themselves] again”* (Participant 11). Some participants further specified they felt hopeful the treatment program would help to decrease their reliance on medication for anxiety support.

“*…I really want to be able to deal with the anxiety or manage the anxiety without medication. I know this is the first step for that, so I'm excited about that”* (Participant 4).

#### “Out of sight, out of mind”

Despite feelings of hopefulness and excitement about beginning the treatment program, many participants forgot that they were even on the waitlist, indicating that it was “*out of sight, out of mind”* (Participant 2) (see also Table 3, quote 15). This response seemed to be mediated somewhat by participants' varying levels of clinical need as well as level of distraction due to other events taking place in their lives.

“*But then I'd say in the last 6 weeks, a lot of things in my life have changed. I got a new job…So it's kind of been a whirlwind of time. So at first I was disappointed that I didn't start the program right away. And then my world just kind of became chaos with this job interview situation…[so] I kind of forgot about [being waitlisted] because I was so fixated on the job side of things in my life”* (Participant 9).

#### Nervousness

Even though all participants were eager to begin the program, some expressed additional apprehension or nervousness about starting (Table 3, quote 16). Participants expressed feeling uncertain about how they would cope with the feelings that might come up during the program.

“*I'm nervous because I think self-exploration is always hard for me…it's always a little bit of like, am I going to open a can of worms of something I didn't know that I had to deal with [chuckles]…it's just exhausting, you know, emotionally and mentally”* (Participant 10).

#### Improving the waitlist experience

At the end of the waitlist period, participants made several suggestions for how their waitlist experience could be improved. These suggestions were categorized into three sub-themes: communication, resource provision, and triaging.

#### Communication

While most participants felt comfortable with the level of communication between themselves and the research team, some participants indicated they would have liked to have received additional communication to check-in and remind them of their place on the waitlist. One suggestion for this involved sending more regular email or text-message check-ins that might also serve to generate excitement about beginning the program and help the participant count down until it was their turn to begin (Table 3, quote 17).

#### Resource provision

Similarly, while participants appreciated the resource list provided by the research team, there were some that expressed feeling overwhelmed by needing to reach out to yet another organization. It was suggested that a more direct referral or a warm handoff to another organization might help to mitigate feelings of overwhelm. Another suggestion made was to create a smaller, more focused or personalized list of resources that would be individually tailored to participants' unique needs. A personalized list might keep in mind details such as the participant's level of clinical need, if they were prenatal or postpartum, or living in a rural or urban setting. Some participants also indicated that they would have appreciated more self-guided resources.

“*…just something that I could do kind of in the meantime [like self-guided breathing exercises], as opposed to just kind of waiting… in the feelings that I was feeling, waiting for it to start to learn things. Because, I mean, …it is a good chunk of time to have to wait while you're feeling those feelings”* (Participant 13).

A further suggestion was to, in the future, use self-guided CBT programs such as the one utilized by the current study, as waitlist interventions themselves that participants could access while waiting to begin other treatments (Table 3, quote 18).

#### Triaging

Finally, there were participants that expressed wishing that consideration for their mental health history and/or where they were at in the perinatal period be taken as a way of determining how urgently they required service. Despite knowing the selection for the present study was random, there also appeared to be a general assumption among some participants that their level of clinical need would or should be considered when it came to assigning them to be in the waitlist or treatment condition (see Table 3, quote 19, 20).

## Discussion

The present study makes a novel contribution to the literature by being the first study to explore the unique experiences and needs of perinatal women on the waitlist for a randomized controlled trial intervention for perinatal anxiety. Findings highlight the importance of mental health services during the perinatal period and that the timing in which they are offered is critical. While previous waitlist studies have indicated that participants often felt they needed treatment more urgently than they received it (5, 7, 10), our study highlights the unique timing that must be considered when providing mental health services to perinatal individuals, particularly early on in both the prenatal and postpartum periods. These findings align with a previous study by Vigod and colleagues (35), which found that a Canadian postpartum sample sought mental health support more commonly during the first 3 months of the postpartum period.

When it came to initially seeking mental health support, participants in our study noted that they had difficulty identifying what they were feeling. According to Jorm (56), the term *mental health literacy* is used to describe the level of public knowledge regarding mental disorders. The broader literature supports that lower mental health literacy, specifically around perinatal mental health concerns, amongst healthcare providers and perinatal individuals may impair perinatal individuals' ability to access supports for perinatal anxiety (23, 36, 57, 58). For example, perinatal participants in a qualitative study by Harrison et al. (23) expressed difficulty identifying their symptoms of perinatal anxiety, which impeded their ability to seek supports. Low mental health literacy was not only mentioned by our participants as a barrier to initially seeking help, but was also evident when it came to accessing supports on the resource list provided to participants during the waitlist. Many participants indicated that they felt unable to identify which supports would be most well-suited to their needs. This finding indicates the importance of perinatal education focused on mental health conditions, which may help pregnant and postpartum individuals identify their symptoms and seek appropriate supports. Further to this, healthcare providers should consider taking the time to assist perinatal patients by connecting them with appropriate resources, particularly if they are waitlisted for another service.

While participants in the present study initially expressed overall negative feelings and disappointment about being waitlisted, there were some participants who felt that knowing help would be coming made the six-week wait time more manageable. These results are in keeping with findings from previous non-perinatal mental health and surgical waitlist studies (8–10). Participants in the present study identified coping strategies that they found helpful during the waitlist period, many of which have been reported across the waitlist literature, in particular, peer support, self-help materials, and other professional supports (7, 10). While previous non-perinatal waitlist studies have often cited physical exercise as a coping strategy used while on waitlists, our participants did not identify this, perhaps due to limited physical capabilities depending on where they found themselves in the perinatal period.

Moreover, it must be noted that the current study is also novel in that it is the first to examine the waitlist experiences of a perinatal population seeking mental health support during the COVID-19 pandemic. In keeping with previous literature about perinatal mental health during the COVID-19 pandemic (37), some of our participants identified feeling that there were not enough spaces within remote perinatal services (e.g., online birthing classes) to keep up with demand. Lack of service accessibility and long waitlists may have compounded feelings of isolation, which are commonly experienced by perinatal individuals under non-pandemic circumstances (23, 59). Additionally, the COVID-19 pandemic meant that regular access to formal professional supports or informal peer supports may have been delayed, avoided, or altogether unable to occur. In light of this, healthcare providers should be mindful that perinatal individuals may need additionally triaging while on the waitlist for mental health services and may need additional assistance connecting with resources, depending on their symptoms and level of informal supports.

It follows that our findings highlight the value of informal peer supports during the waitlist period. Participants identified that connecting with other mothers who understood what they were going through helped to normalize and validate their experiences, which aligns with previous qualitative findings (23, 60). Qualitative research has found that perinatal people experiencing mental health problems often feel shame or guilt for not having a ‘normal' perinatal experience (22, 61, 62), thereby highlighting the critical role that peer supports can play in reducing stigma and encouraging mental health help-seeking in the perinatal period (23, 24, 59). Further to this, healthcare providers should recognize the value of informal peer supports and consider how they might further facilitate peer connections (both on and offline) during waitlist periods.

Furthermore, previous studies have identified additional recommendations on how to improve the waitlist experience, including: (a) periodic updates from healthcare providers, (b) transparency regarding the prioritization criteria, (c) estimates for the length of wait, and (d) intermediate interventions to help participants through the wait (4, 5). Our study's findings are not only consistent with these recommendations but provide novel and valuable suggestions to the literature for vulnerable populations, specifically for perinatal women. Recommended improvements that emerged from the data included (a) more periodic email or text-message updates from providers, (b) intermediate interventions during the waitlist period, such as additional self-help resource recommendations, direct referrals to another service provider, or using online self-guided programs as waitlist interventions themselves, and (c) consideration for clinical need and/or where individuals are in the perinatal period. In the current study, participants also expressed appreciation for the relatively short, definitive time period spent on the waitlist, in keeping with previous findings that recommend providing estimates for the length of wait (5, 6). Researchers conducting future studies with perinatal populations should take the above recommendations into consideration when utilizing waitlist conditions; setting definitive wait times, checking in regularly with participants, and providing appropriate intermediate interventions may help to build and maintain a positive rapport with the research team and mitigate potential mental health declines during the waitlist period.

### Limitations

Study findings may be limited due to the lack of diversity among participants, with the majority of participants being of European background, heterosexual, college or university educated, and in a relationship with a common-law partner or spouse. Furthermore, the OPA program was offered online and therefore required participants to have a stable Internet connection, which may have prevented certain low-income and marginalized groups from participating. Exclusion criteria prevented individuals experiencing substance abuse disorders, diagnoses of schizophrenia or bipolar disorder, and current suicidality from participating in the study, which additionally limited the diversity of participants.

Study findings may also be limited by the definitive and relatively short duration of the waitlist period. While this may limit the generalizability of findings, the present study offers valuable preliminary insight into the experiences of perinatal people waitlisted for mental health services. The present study may serve to inform future research examining the effects of variable wait-times and differing levels of clinician contact on waitlist experiences.

## Conclusions

When an individual experiences anxiety, it can be difficult to seek assistance. The mental health effects of being waitlisted may be magnified for perinatal populations given the number of rapidly occurring changes that they experience. The present study examined how perinatal individuals are uniquely affected by waitlists, specifically within the context of a randomized controlled trial during the COVID-19 pandemic, and offers improvements for future waitlist conditions. Recommendations for service providers include ensuring that perinatal clients are connected with mental health resources pre-emptively to reduce time spent on waitlists; offering increased communication and periodic updates during the waitlist period; providing more direct referrals to intermediate interventions; facilitating increased peer-support during the waitlist period; and ensuring that clients have support during the most critical periods early in the prenatal and postpartum periods. Results of this study may also help to inform healthcare providers of who warrants earlier interventions. Furthermore, our findings provide a nuanced understanding of the experiences of perinatal women struggling with anxiety, allowing the potential for more tailored approaches to mental health support for those awaiting services and directions for improved support. Future research should further explore and evaluate support strategies for perinatal individuals who are waitlisted for mental health services. In particular, a deeper exploration of strategies to facilitate peer support during the perinatal period is warranted and may help to relieve some of the demand placed on mental health providers around waitlists. Additionally, it would be of value to explore the potential for peer support to attenuate deteriorations in mental health during the waitlist period.

## Data availability statement

The raw data supporting the conclusions of this article will be made available by the authors, without undue reservation.

## Ethics statement

The studies involving human participants were reviewed and approved by University of Manitoba Research Ethics Board. The patients/participants provided their written informed consent to participate in this study.

## Author contributions

KR conceptualized and designed the study. KR, GA, and PF obtained funding for this project. AK, KR, MG, MH, SP, and TP conducted qualitative interviews. AK and MH reviewed and edited qualitative interview transcripts. AK, KR, and MH analyzed qualitative interview transcripts and created the thematic framework. MG and MH oversaw quantitative data collection. MH conducted quantitative data analysis. AK and KP conducted the literature search. AK wrote the first draft of the manuscript. GA, KR, KP, and MH provided feedback and edited the manuscript. All authors approved the final manuscript.

## Funding

We acknowledge funding received from Research Manitoba (K Reynolds, PI).

## Conflict of interest

The authors declare that the research was conducted in the absence of any commercial or financial relationships that could be construed as a potential conflict of interest.

## Publisher's note

All claims expressed in this article are solely those of the authors and do not necessarily represent those of their affiliated organizations, or those of the publisher, the editors and the reviewers. Any product that may be evaluated in this article, or claim that may be made by its manufacturer, is not guaranteed or endorsed by the publisher.
